# Bifurcation Analysis in Models for Vector-Borne Diseases with Logistic Growth

**DOI:** 10.1155/2014/195864

**Published:** 2014-03-25

**Authors:** Guihua Li, Zhen Jin

**Affiliations:** Department of Mathematics, North University of China, Taiyuan, Shanxi 030051, China

## Abstract

We establish and study vector-borne models with logistic and exponential growth of vector and host populations, respectively. We discuss and analyses the existence and stability of equilibria. The model has backward bifurcation and may have no, one, or two positive equilibria when the basic reproduction number *R*_0_ is less than one and one, two, or three endemic equilibria when *R*_0_ is greater than one under different conditions. Furthermore, we prove that the disease-free equilibrium is stable if *R*_0_ is less than 1, it is unstable otherwise. At last, by numerical simulation, we find rich dynamical behaviors in the model. By taking the natural death rate of host population as a bifurcation parameter, we find that the system may undergo a backward bifurcation, saddle-node bifurcation, Hopf bifurcation, Bogdanov-Takens bifurcation, and cusp bifurcation with the saturation parameter varying. The natural death rate of host population is a crucial parameter. If the natural death rate is higher, then the host population and the disease will die out. If it is smaller, then the host and vector population will coexist. If it is middle, the period solution will occur. Thus, with the parameter varying, the disease will spread, occur periodically, and finally become extinct.

## 1. Introduction

The dynamical modeling and studies of vector-borne disease have been in many literatures. The earliest studies originated from the Ross-Macdonald model for malaria [1] in 1911 and major extensions are described in Macdonald's 1957 book [2]. For malaria, there aremany literature [3–7]. Research literatures on dengue fever have [8–12], and so forth. For West Nile virus (WNV), there are also many literatures, [13–18], and so forth. Here, we classify vector population as susceptible (*S*_*v*_) and infected (*I*_*v*_) and classify the host population as susceptible (*S*_*h*_), infected (*I*_*h*_), and recovered *R*_*h*_. In these literatures, birth function of susceptible host and vector population and incidence rate are taken as Table 1. Parameters involved in Table 1 for the dynamics of vector-borne diseases are in Table 2. For malaria, dengue fever, and West Nile virus, the three typical vector-borne diseases, the dynamics of most of the related models can be characterized by the reproduction number, but some compartmental models for these three mosquito-borne diseases undergo a backward bifurcation, an important feature of mosquito-borne diseases.

For compartmental models for malaria, dengue fever, and WNV, different forms of birth functions have been used; we summarize and find that the logistic birth function which describes the self-limiting growth of a host population was rarely considered. In the paper, we will build the following model and analyse the dynamic behaviors:

(1) $$\begin{array}{c} \frac{dS_{h}}{dt}=rS_{h}\left(1-\frac{N_{h}}{K}\right)-\frac{\beta_{h}I_{v}S_{h}}{a_{1}N_{h}+a_{2}N_{v}}-d_{h}S_{h},\\ \frac{dI_{h}}{dt}=\frac{\beta_{h}I_{v}S_{h}}{a_{1}N_{h}+a_{2}N_{v}}-\left(\gamma_{h}+d_{h}+\alpha_{h}\right)I_{h},\\ \frac{dR_{h}}{dt}=\gamma_{h}I_{h}-d_{h}R_{h},\\ \frac{dN_{h}}{dt}=rS_{h}\left(1-\frac{N_{h}}{K}\right)-d_{h}N_{h}-\alpha I_{h},\\ \frac{dS_{v}}{dt}=d_{v}S_{v}+\left(1-p\right)d_{v}I_{v}-\frac{\beta_{v}I_{h}S_{v}}{a_{1}N_{h}+a_{2}N_{v}}-d_{v}S_{v},\\ \frac{dI_{v}}{dt}=pd_{v}I_{v}+\frac{\beta_{v}I_{h}S_{v}}{a_{1}N_{h}+a_{2}N_{v}}-d_{v}I_{v}. \end{array}$$

In the model (1), the host and vector populations satisfy the following equations, respectively:

(2) $$\begin{array}{c} \frac{dN_{h}}{dt}=rS_{h}\left(1-\frac{N_{h}}{K}\right)-d_{h}N_{h}-\alpha I_{h},\\ \frac{dN_{v}}{dt}=0. \end{array}$$

It means that the total number of the vector populations is a constant and denotes *N*_*v*_ = *N*_*v*0_. Furthermore, using the relations *S*_*h*_ + *I*_*h*_ + *R*_*h*_ = *N*_*h*_ and *S*_*v*_ = *N*_*v*_ − *I*_*v*_, we lead to the following four-dimensional nonlinear system of ODEs:

(3) $$\begin{array}{c} \frac{dS_{h}}{dt}=rS_{h}\left(1-\frac{N_{h}}{K}\right)-\frac{\beta_{h}I_{v}S_{h}}{a_{1}N_{h}+a_{2}N_{v0}}-d_{h}S_{h},\\ \frac{dI_{h}}{dt}=\frac{\beta_{h}I_{v}S_{h}}{a_{1}N_{h}+a_{2}N_{v0}}-\left(\gamma_{h}+d_{h}+\alpha_{h}\right)I_{h},\\ \frac{dN_{h}}{dt}=rS_{h}\left(1-\frac{N_{h}}{K}\right)-d_{h}N_{h}-\alpha I_{h},\\ \frac{dI_{v}}{dt}=pd_{v}I_{v}+\frac{\beta_{v}I_{h}\left(N_{v0}-I_{v}\right)}{a_{1}N_{h}+a_{2}N_{v0}}-d_{v}I_{v}. \end{array}$$

One can verify that the positive cone

(4) $$\begin{array}{c} \Omega =\left\{0\leq S_{h}+I_{h}+R_{h}\leq \left(1-\frac{d_{h}}{r}\right)K,0\leq S_{v}+I_{v}\leq N_{v0}\right\} \end{array}$$

is positive invariant.

The remainder of this paper is organized as follows. In Section 2, we consider the number and existence of equilibria.  Section 3 studies local stability of the disease-free equilibrium and gives some examples to explain the existence of endemic equilibria and bifurcation with the basic reproduction number changing. The paper concludes with a brief discussion in Section 4.

## 2. Existence of Equilibria

To study the existence of equilibria, let the right side of each of the four differential equations be equal to zero in system (3), obtaining the following equations:

(5) $$\begin{array}{c} rS_{h}\left(1-\frac{N_{h}}{K}\right)-\frac{\beta_{h}I_{v}S_{h}}{a_{1}N_{h}+a_{2}N_{v0}}-d_{h}S_{h}=0,\\ \frac{\beta_{h}I_{v}S_{h}}{a_{1}N_{h}+a_{2}N_{v0}}-\left(\gamma_{h}+d_{h}+\alpha_{h}\right)I_{h}=0,\\ rS_{h}\left(1-\frac{N_{h}}{K}\right)-d_{h}N_{h}-\alpha_{h}I_{h}=0,\\ pd_{v}I_{v}+\frac{\beta_{v}I_{h}\left(N_{v0}-I_{v}\right)}{a_{1}N_{h}+a_{2}N_{v0}}-d_{v}I_{v}=0. \end{array}$$

From the above equations, one can verify that system (3) has a disease-free equilibrium at *E*_0_ : (*S*_*h*0_, *I*_*h*0_, *N*_*h*0_, *I*_*v*0_) = (*K*(1 − *d*_*h*_/*r*), 0, *K*(1 − *d*_*h*_/*r*), 0). The linear stability of *E*_0_ is governed by the basic reproduction number *R*_0_ which can be found from the next generation matrix [19] for the system. Noting that the model has infected populations, namely, *I*_*h*_ and *I*_*v*_, it follows that, using the notation of van den Driessche and Watmough [20], the matrices *F* and *V*, for the new infection terms and the remaining transfer terms, respectively, are given in partitioned form by

(6) $$\begin{array}{c} F=\left(\begin{array}{cc} F_{1} & 0\\ F_{2} & 0 \end{array}\right),\;\;V=\left(\begin{array}{cc} V_{1} & 0\\ 0 & V_{2} \end{array}\right), \end{array}$$

where

(7) $$\begin{array}{c} F_{1}=\left(\begin{array}{cc} 0 & \frac{\beta_{h}S_{h0}}{a_{1}N_{h0}+a_{2}N_{v0}}\\ \frac{\beta_{v}N_{v0}}{a_{1}N_{h0}+a_{2}N_{v0}} & 0 \end{array}\right),\\ V_{1}=\left(\begin{array}{cc} d_{h}+\gamma_{h}+\alpha_{h} & 0\\ 0 & \left(1-p\right)d_{v} \end{array}\right). \end{array}$$

Here, *F* is a nonnegative matrix of rank 2 and *V* is nonsingular. It is easy to show that, at steady state, the spectral radius (dominant eigenvalue) of the nonnegative matrix *FV* ^−1^, denoted by *ρ*(*FV* ^−1^), is equal to *ρ*(*F*_1_ *V*_1_^−1^); hence,

(8) R0=(βhSh0(dh+γh+αh)(a1Nh0+a2Nv0) ×βvNv0(1−p)dv(a1Nh0+a2Nv0))1/2=βhβvSh0Nv0(a1Nh0+a2Nv0)2(1−p)dv(dh+γh+αh).

One can see that *R*_0_ is the geometric mean of

(9) $$\begin{array}{c} \frac{\beta_{h}S_{h0}}{\left(d_{h}+\gamma_{h}+\alpha_{h}\right)\left(a_{1}N_{h0}+a_{2}N_{v0}\right)},\\ \frac{\beta_{v}N_{v0}}{\left(1-p\right)d_{v}\left(a_{1}N_{h0}+a_{2}N_{v0}\right)}, \end{array}$$

the number of new diseased generated in both the host and vector population, respectively. The first part is the number of host infections caused by one infected vector; the second part is the number of vector infections caused by one infected host. Discussion in detail on basic reproductive number of host-vector disease models see literature [16].

In order to find positive equilibria, it follows from the first and last two equations of (5), we have

(10) $$\begin{array}{c} S_{h}=\frac{\left(d_{h}N_{h}+\alpha_{h}I_{h}\right)K}{r\left(K-N_{h}\right)},\\ I_{h}=\frac{\left(1-p\right)d_{v}I_{v}\left(a_{1}N_{h}+a_{2}N_{v0}\right)}{\beta_{v}\left(N_{v0}-I_{v}\right)},\\ I_{v}=\frac{\left(\left(r-d_{h}\right)K-rN_{h}\right)\left(a_{1}N_{h}+a_{2}N_{v0}\right)}{K\beta_{h}}. \end{array}$$

Substituting (10) into the second equation of (5), that is, if an endemic equilibrium exists, its *N*_*h*_ coordinate satisfies

(11) $$\begin{array}{c} F\left(n_{h}\right)=A_{3}N_{h}^{3}+A_{2}N_{h}^{2}+A_{1}N_{h}+A_{0}=0, \end{array}$$

where

(12) A3=a1r[dv(dh+γh)(1−p)a1+βvdh]>0,A2=dv(1−p)K[αh(r−dh)−δhr]a12  +[2dvr(dh+γh)(1−p)Nv0a2   −dhβvK(r−dh)]a1+dhrβvNv0a2,A1=[dvr(dh+γh)(1−p)Nv0a22   +2Kdv(1−p)(α1(r−dh)−δhr)a1a2   −dhβvK(r−dh)a2+dhβhβvK]Nv0,A0=dvK(1−p)[αh(r−dh)−δhr]Nv02a22<0.

*N*_*h*_ should satisfy 0 < *N*_*h*_ < *N*_*h*0_ = *K*(1 − *d*_*h*_/*r*) to ensure a positive equilibrium. Then we will consider the existence of positive roots for (11) from the following two cases.

### 2.1. Case 1: *a*_1_ ≠ 0, *a*_2_ = 0

In this case, system (3) for vector-borne diseases is reduced to

(13) $$\begin{array}{c} \frac{dS_{h}}{dt}=rS_{h}\left(1-\frac{N_{h}}{K}\right)-\frac{\beta_{h}I_{v}S_{h}}{N_{h}}-d_{h}S_{h},\\ \frac{dI_{h}}{dt}=\frac{\beta_{h}I_{v}S_{h}}{N_{h}}-\left(\gamma_{h}+d_{h}+\alpha_{h}\right)I_{h},\\ \frac{dN_{h}}{dt}=rS_{h}\left(1-\frac{N_{h}}{K}\right)-d_{h}N_{h}-\alpha_{h}I_{h},\\ \frac{dI_{v}}{dt}=pd_{v}I_{v}+\frac{\beta_{v}I_{h}\left(N_{v0}-I_{v}\right)}{N_{h}}-d_{v}I_{v}. \end{array}$$

For system (13), *N*_*h*_ satisfies

(14) $$\begin{array}{c} F_{1}\left(N_{h}\right)=B_{2}N_{h}^{2}+B_{1}N_{h}+B_{0}=0, \end{array}$$

where

(15) B2=r(dh+γh)dv+rdhβv>0,B1=−K(1−p)[(dh+γh)r+dhαh]dv  −dhβv(r−dh)K<0,B0=dhKβhβvNv0>0.

Now we consider the case 0 ≤ *p* < 1. One can see that it is the number of roots in the interval (0, *K*(1 − *d*_*h*_/*r*)) that determines the number of positive equilibria for model (13). Note

(16) $$\begin{array}{c} F_{1}\left(0\right)=d_{h}K\beta_{h}\beta_{v}N_{v0}>0,\\ F_{1}\left(K\left(1-\frac{d_{h}}{r}\right)\right)=\delta_{h}d_{h}\left(1-p\right)d_{2}K\left(1-\frac{d_{h}}{r}\right)\left(R_{0}^{2}-1\right). \end{array}$$

If *R*_0_ < 1, *F*_1_(*K*(1 − *d*_*h*_/*r*)) < 0, *F*_1_(*N*_*h*_) = 0 has a unique root in (0, *K*(1 − *d*_*h*_/*r*)). If *R*_0_ > 1, *F*_1_(*K*(1 − *d*_*h*_/*r*)) > 0, then there may be two positive equilibria or no positive equilibrium. We have the following theorem.

**Theorem 1**

If *R*_0_ < 1, system (13) has a unique positive equilibrium E*(*S*_h_*, *I*_h_*, *N*_h_*, *I*_v_*).

**Theorem 2**

If *R*_0_ > 1, one has the following.

- If −*rB*_1_ ≥ *K*(*r* − *d*_*h*_)*B*_2_, then system (13) has no endemic equilibrium.
- If −*rB*_1_ < *K*(*r* − *d*_*h*_)*B*_2_, then
  - when *B*_1_^2^ < 4*B*_2_ *B*_0_, system (13) has no endemic equilibrium;
  - when *B*_1_^2^ = 4*B*_2_ *B*_0_, system (13) has a unique repeated endemic equilibrium E** = (*S*_h_**, *I*_h_**, *N*_h_**, *I*_v_**);
  - when *B*_1_^2^ > 4*B*_2_ *B*_0_, system (13) has two endemic equilibria *E*_1_*(*S*_*h*_^∗1^, *I*_*h*_^∗1^, *N*_*h*_^∗1^, *I*_*v*_^∗1^), *E*_2_*(*S*_*h*_^∗2^, *I*_*h*_^∗2^, *N*_*h*_^∗2^, *I*_*v*_^∗2^), where *N*_*h*_^∗1^ < *N*_h_** < *N*_*h*_^∗2^.

### 2.2. Case 2: *a*_1_ ≠ 0, *a*_2_ ≠ 0

For *F*(*N*_*h*_) of system (3),

(17) F(0)=A0<0,F(K(1−dhr))=Kdh(dh+αh+γh)(1−p)dv×(a1Nh0+a2Nv0)2(R02−1).

If *R*_0_ < 1, *F*(*K*(1 − *d*_*h*_/*r*)) < 0 and *R*_0_ > 1, *F*(*K*(1 − *d*_*h*_/*r*)) > 0. Then we will analyze the existence of positive roots of *F*(*N*_*h*_) = 0. The derivative of *F*(*N*_*h*_) is

(18) $$\begin{array}{c} F^{\prime }\left(N_{h}\right)=3A_{3}N_{h}^{2}+2A_{2}N_{h}+A_{1}. \end{array}$$

Denote

(19) $$\begin{array}{c} N_{h1}=\frac{A_{2}-\sqrt{A_{2}^{2}-3A_{3}A_{1}}}{3A_{3}},\\ N_{h2}=\frac{A_{2}+\sqrt{A_{2}^{2}-3A_{3}A_{1}}}{3A_{3}}. \end{array}$$

Then we have the following theorem.

**Theorem 3**

If *R*_0_ < 1, one has the following.

- System (3) has no endemic equilibrium, if any one of the following conditions holds:
  1. *A*_2_ > 0 and *A*_1_ < 0,
  2. *A*_2_ < 0, *A*_1_ > 0, and *K*(1 − *d*_*h*_/*r*) < *N*_*h*0_,
  3. *A*_2_ < 0, *A*_1_ > 0, *K*(1 − *d*_*h*_/*r*) > *N*_*h*0_, and *F*(*N*_*h*1_) > 0.
- System (3) has two endemic equilibria, if *A*_2_ < 0, *A*_1_ > 0, *K*(1 − *d*_*h*_/*r*) > *N*_*h*0_, and *F*(*N*_*h*1_) < 0.

**Theorem 4**

If *R*_0_ > 1, one has the following.

- System (3) has a unique endemic equilibrium, if any one of the following conditions holds:
  1. *A*_2_ > 0 and *A*_1_ < 0;
  2. *A*_2_ < 0, *A*_1_ > 0, *A*_2_^2^ ≤ 3*A*_3_ *A*_1_;
  3. *A*_2_ < 0, *A*_1_ > 0, *A*_2_^2^ > 3*A*_3_ *A*_1_, and *K*(1 − *d*_*h*_/*r*) < *N*_*h*0_;
  4. *A*_2_ < 0, *A*_1_ > 0, *A*_2_^2^ > 3*A*_3_ *A*_1_, *K*(1 − *d*_*h*_/*r*) > *N*_*h*0_, *F*(*N*_*h*1_) < 0;
  5. *A*_2_ < 0, *A*_1_ > 0, *A*_2_^2^ > 3*A*_3_ *A*_1_, *K*(1 − *d*_*h*_/*r*) > *N*_*h*0_, *F*(*N*_*h*1_) > 0, and *F*(*N*_*h*2_) > 0.
- System (3) has three endemic equilibria, if *A*_2_ < 0, *A*_1_ > 0, *A*_2_^2^ > 3*A*_3_ *A*_1_, *K*(1 − *d*_*h*_/*r*) > *N*_*h*0_, *F*(*N*_*h*1_) > 0, and *F*(*N*_*h*2_) < 0.

It is easy to prove Theorem 4, we will omit the proof. In the following part, we will give two examples to show changes of equilibria with the basic reproduction number *R*_0_ by numerical simulation when *a*_2_ = 0 and *a*_2_ ≠ 0.

**Example 5**

**Figure 1:**
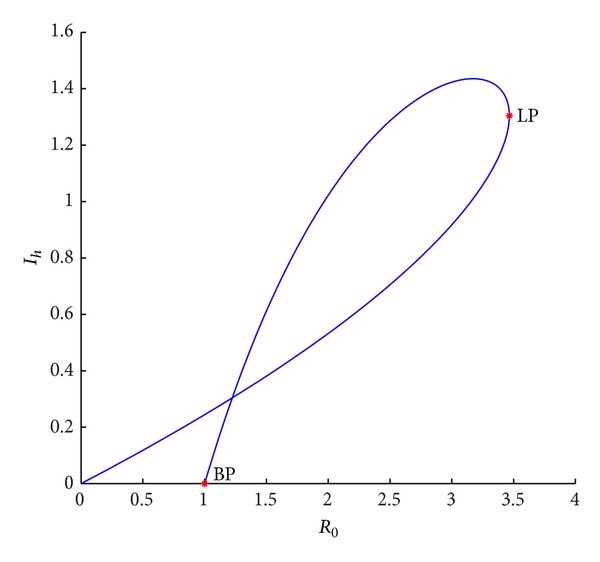
Bifurcation curves in (*R*_0_, *I*_*h*_) plane when *r* = 0.8, *K* = 10, *β*_1_ = 1, *a*_1_ = 1, *a*_2_ = 0, *d*_*h*_ = 0.036, *γ*_*h*_ = 0.06, *α*_*h*_ = 0.36, *p* = 0.007, *d*_*v*_ = 0.06, *β*_2_ = 0.26, and *N*_*v*_ = 2.

Fix parameters *r* = 0.8, *K* = 10, *a*_1_ = 1, *a*_2_ = 0, *d*_*h*_ = 0.036, *γ*_*h*_ = 0.06, *α*_*h*_ = 0.36, *p* = 0.007, *d*_*v*_ = 0.06, *β*_2_ = 0.26, and *N*_*v*_ = 2.  Figure 1 shows that there is always a unique endemic equilibrium when *R*_0_ < 1. As *R*_0_ increases, the equilibrium will be keep. If *R*_0_ equals one, a new positive equilibrium is branched from the disease-free equilibrium. If *R*_0_ continue to be increased, the two positive equilibria may be repeated and then disappeared if *R*_0_ exceeds a certain value, that is, there are two positive equilibria or no positive equilibrium when *R*_0_ > 1 if *a*_2_ = 0.

**Example 6**

**Figure 2:**
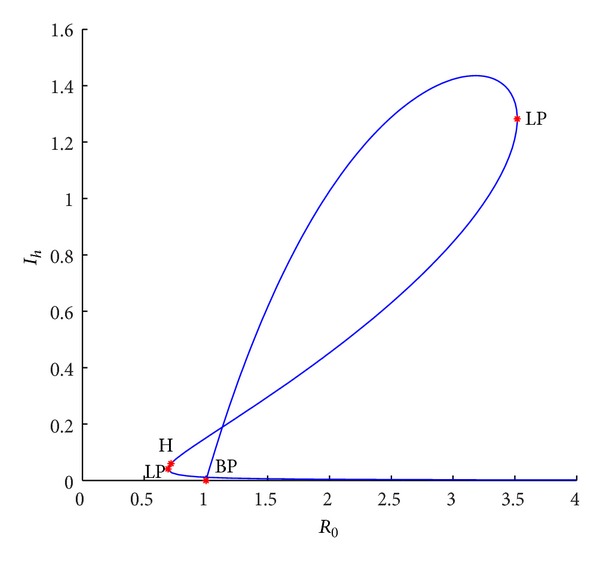
Bifurcation curves in (*R*_0_, *I*_*h*_) plane when *r* = 0.8, *K* = 10, *β*_1_ = 1, *a*_1_ = 1, *a*_2_ = 0.1, *d*_*h*_ = 0.036, *γ*_*h*_ = 0.06, *α*_*h*_ = 0.36, *p* = 0.007, *d*_*v*_ = 0.06, *β*_2_ = 0.26, and *N*_*v*_ = 2.

**Figure 3:**
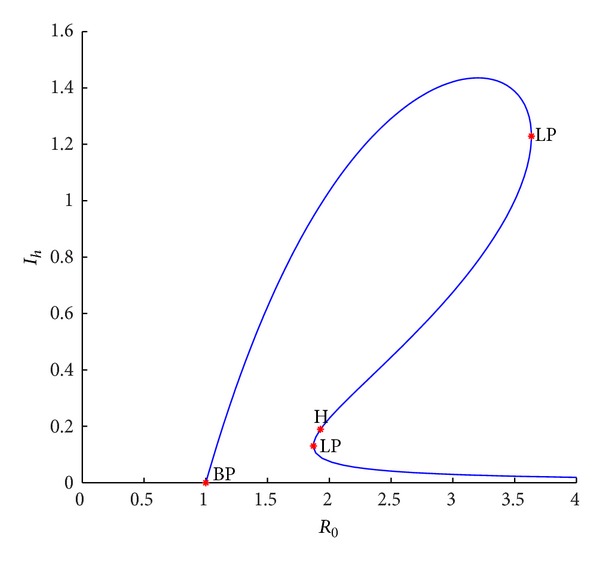
Bifurcation curves in (*R*_0_, *I*_*h*_) plane when *r* = 0.8, *K* = 10, *β*_1_ = 1, *a*_1_ = 1, *a*_2_ = 0.3, *d*_*h*_ = 0.036, *γ*_*h*_ = 0.06, *α*_*h*_ = 0.36, *p* = 0.007, *d*_*v*_ = 0.06, *β*_2_ = 0.26, and *N*_*v*_ = 2.

Take *r*, *K*, *a*_1_, *d*_*h*_, *γ*_*h*_, *α*_*h*_, *p*, *d*_*v*_, *β*_2_, and *N*_*v*_ as same values of Example 5 and *a*_2_ = 0.1. From Figure 2, we find that the number of positive equilibria is from zero to two with *R*_0_ increasing when *R*_0_ < 1. If *R*_0_ is more than one, it is found that the two positive equilibria still be keep and there is a new endemic one to be branched from the disease-free equilibrium. Now system (3) has three endemic equilibria. Continuing to increase *R*_0_, we find that the larger two equilibria will be repeated and then disappear and the smaller equilibrium always be keep. Let *a*_2_ = 0.3; from Figure 3, it can be obtained that there is no positive equilibrium when *R*_0_ < 1. With *R*_0_ increasing, a positive equilibrium is branched from the disease-free one; then three ones appear and the larger two ones disappear and the smaller one lasts.

## 3. Stability and Bifurcation Analysis

Then we study the stability of the steady states of system (3) and possible bifurcations. Firstly, we discuss the stability of disease-free equilibrium *E*_0_. At this equilibrium point, the Jacobian matrix is

(20) $$\begin{array}{c} J_{0}=\left(\begin{array}{cccc} 0 & 0 & a_{13} & a_{14}\\ 0 & -\delta_{h} & 0 & -a_{14}\\ d_{h} & -\alpha_{h} & a_{13}-d_{h} & 0\\ 0 & a_{42} & 0 & -\left(1-p\right)d_{v} \end{array}\right), \end{array}$$

where

(21) a13=−r(1−dhr)<0,a14=−βhK(1−dh/r)a1K(1−dh/r)+a2Nv0<0,a42=βvNv0a1K(1−dh/r)+a2Nv0>0.

The characteristic polynomial of (20) is

(22) [λ2+(−a13+dh)λ−a13dh] ×[λ2+(δh+(1−p)dv)λ+a42a14+δh(1−p)dv] =0.

It means that

(23) $$\begin{array}{c} \lambda^{2}+\left(-a_{13}+d_{h}\right)\lambda -a_{13}d_{h}=0, \end{array}$$

or

(24) λ2+(δh+(1−p)dv)λ+a42a14+δh(1−p)dv =λ2+(δh+(1−p)dv)λ+δh(1−p)dv(1−R0) =0.

The roots of the two quadratic polynomial have negative real parts if and only if their coefficients are positive; that is, *R*_0_ < 1. Therefore, the disease-free equilibrium *E*_0_ is always locally asymptotically stable when *R*_0_ < 1, and it is unstable if *R*_0_ > 1.

For any positive endemic equilibrium *E*_*i*_, the Jacobian matrix of (3) becomes

(25) $$\begin{array}{c} J_{i}=\left(\begin{array}{cccc} 0 & 0 & -\frac{rS_{h}}{K}+a_{23} & -a_{14}\\ a_{21} & -\left(d_{h}+\gamma_{h}+\alpha_{h}\right) & -a_{23} & a_{14}\\ d_{h}+a_{21} & -\alpha_{h} & -d_{h}-rS_{h}/K & 0\\ 0 & a_{42} & -a_{43} & -a_{44} \end{array}\right), \end{array}$$

where

(26) $$\begin{array}{c} a_{14}=\frac{\beta_{h}S_{h}}{a_{1}N_{h}+a_{2}N_{v0}},\;\;a_{21}=\frac{\beta_{h}I_{v}}{a_{1}N_{h}+a_{2}N_{v0}},\\ a_{23}=\frac{a_{1}\beta_{h}I_{v}S_{h}}{{\left(a_{1}N_{h}+a_{2}N_{v0}\right)}^{2}},\;\;a_{42}=\frac{\beta_{v}\left(N_{v0}-I_{v}\right)}{a_{1}N_{h}+a_{2}N_{v0}},\\ a_{43}=\frac{a_{1}\beta_{v}I_{h}\left(N_{v0}-I_{v}\right)}{{\left(a_{1}N_{h}+a_{2}N_{v0}\right)}^{2}},\\ a_{44}=\left(1-p\right)d_{v}+\frac{\beta_{v}I_{h}}{a_{1}N_{h}+a_{2}N_{v0}}. \end{array}$$

Here, it is difficult to determine a sign for the real part of eigenvalue of *J*_*i*_(*E*_*i*_), so we will give the dynamical behaviors for system (3) by numerical simulation.

**Example 7**

**Figure 4:**
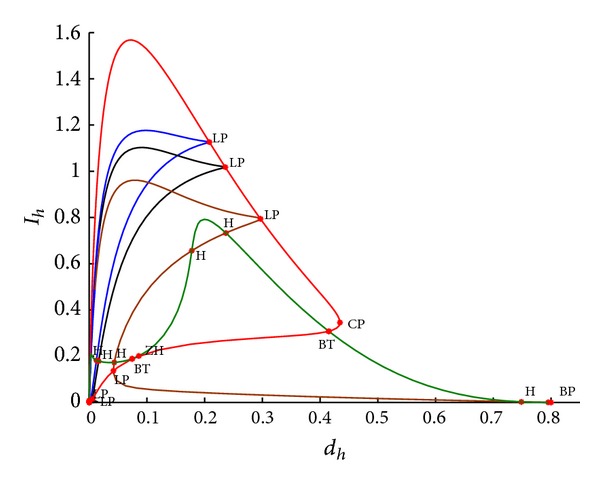
Bifurcation curves in (*d*_*h*_, *I*_*h*_) plane when *r* = 0.8, *K* = 10, *β*_1_ = 1, *a*_1_ = 1, *a*_2_ = 0,0.1,0.3, *d*_*h*_ = 0.036, *γ*_*h*_ = 0.06, *α*_*h*_ = 0.36, *p* = 0.007, *d*_*v*_ = 0.06, *β*_2_ = 0.26, and *N*_*v*_ = 2.

Fix parameters *r* = 0.8, *K* = 10, *a*_1_ = 1, *a*_2_ = 0, *d*_*h*_ = 0.036, *γ*_*h*_ = 0.06, *α*_*h*_ = 0.36, *p* = 0.007, *d*_*v*_ = 0.06, *β*_2_ = 0.26, and *N*_*v*_ = 2. In Figure 1, we find that a unique positive equilibrium is unstable when *R*_0_ < 1. With *R*_0_ increasing and exceeding one, the unstable positive equilibrium still be keep and a new positive one branching from the disease-free one is stable. After *R*_0_ is more than a threshold, the two equilibria will disappear, and all of trajectories tend to equilibrium *E*(*S*_*h*_, *I*_*h*_, *R*_*h*_, *S*_*v*_, *I*_*v*_) = (0,0, 0, *N*_*v*0_, 0), which means that the host population will be extinct If we fix *R*_0_ and let *d*_*h*_ change, we find that Hopf bifurcation maybe occurs from the blue curve of Figure 4 for system (13).

**Example 8**

Fix parameters *r* = 0.8, *K* = 10, *a*_1_ = 1, *β*_*h*_ = 1, *γ*_*h*_ = 0.06, *α*_*h*_ = 0.36, *p* = 0.007, *d*_*v*_ = 0.06, *β*_2_ = 0.26, and *N*_*v*_ = 2. Then we draw the bifurcation figure with *d*_*h*_ changed when *a*_2_ = 0,0.1,0.3, respectively. We use blue, black, and brown curves to represent the bifurcation curves when *a*_2_ = 0,0.1,0.3. In Figure 4, we firstly choose *d*_*h*_ as bifurcation parameter and draw the bifurcation curve (blue (*a*_2_ = 0), black (*a*_2_ = 0.1), and brown curves (*a*_2_ = 0.3)) *I*_*h*_ with *d*_*h*_. When *a*_2_ = 0, we find that *R*_0_ is always more than 1 no matter what the value of *d*_*h*_ is, which means that system (3) has no or two positive equilibria. From the blue curve of Figure 4, we can see that there are a limit point (LP) at *d*_*h*_ = 0.208198 and a Hopf point (H) at *d*_*h*_ = 0.012541 whose first Lyapunov coefficient is 4.560482*e* − 001. When *a*_2_ = 0.1, it is found that two limit points at *d*_*h*_ = 0.235734 and 0.005803 and a Hopf point at *d*_*h*_ = 0.016349 whose first Lyapunov coefficient is 6.142143*e* − 001 for system (3). This is consistent with the existence of the positive equilibrium. If we take *a*_2_ = 0.3, by numerical simulation, we see that there are two limit points at *d*_*h*_ = 0.296694 and 0.041986, two neutral saddle points at *d*_*h*_ = 0.236797 and 0.177735, and two Hopf points at *d*_*h*_ = 0.043395 (first Lyapunov coefficient 5.036862*e* + 000) and *d*_*h*_ = 0.748616 (first Lyapunov coefficient −1.827258*e* − 001). Then take a limit point as initial point and *d*_*h*_ and *a*_2_ as bifurcation parameters and obtain a fold curve (red curve in Figure 4). In the fold curve, there are a cusp bifurcation point at *d*_*h*_ = 0.434317, *a*_2_ = 0.631081, and two Bogdanov-Takens bifurcation points at *d*_*h*_ = 0.414918, *a*_2_ = 0.608913 and *d*_*h*_ = 0.074370, *a*_2_ = 0.356609. At the cusp bifurcation point two branches of saddle-node bifurcation curve meet tangentially, forming a semicubic parabola. For nearby parameter values, the system has three equilibria which collide and disappear pairwise via the saddle-node bifurcations. For the Bogdanov-Takens (BT) bifurcation nearby parameter values, the system has two equilibria which collide and disappear via a saddle-node bifurcation. In the end, take any one of Bogdanov-Takens bifurcation points as initial point and draw Hopf bifurcation curve (the green curve of Figure 4). It shows that there is a Zero-Neutral Saddle (ZH) at *d*_*h*_ = 0.085991 and *a*_2_ = 0.370274. Thus, we can obtain that all of points being saddle between two Bogdanov-Takens bifurcation points and Hopf points locate on both sides of Bogdanov-Takens bifurcation points from Hopf bifurcation curve of Figure 4.

In the following example, we will analyse how to change the state variables *I*_*h*_ and *I*_*v*_ with time *t*.

**Example 9**

**Figure 5:**
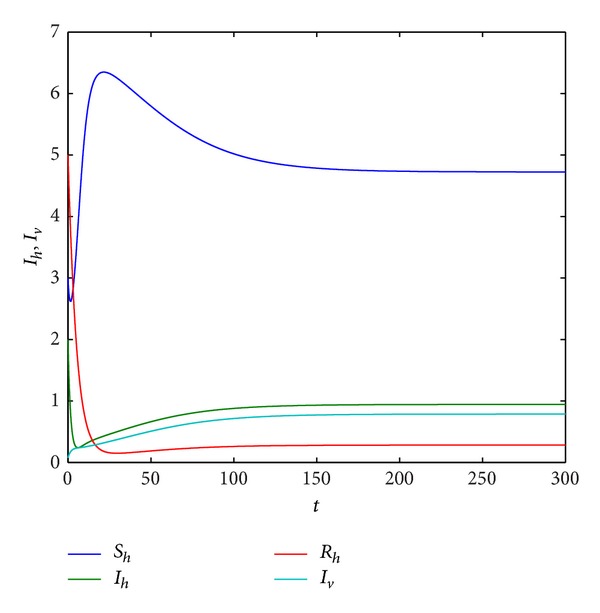
Trajectory picture with time *t* when *r* = 0.8, *K* = 10, *β*_1_ = 1, *a*_1_ = 1, *a*_2_ = 0.3, *d*_*h*_ = 0.2, *γ*_*h*_ = 0.06, *α*_*h*_ = 0.36, *p* = 0.007, *d*_*v*_ = 0.06, *β*_2_ = 0.26, and *N*_*v*_ = 2.

**Figure 6:**
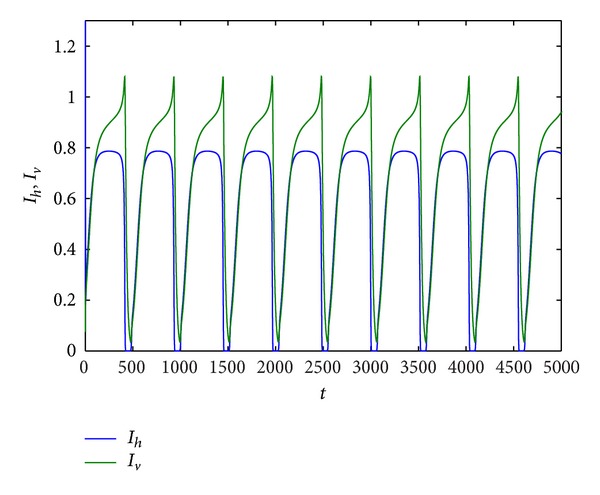
Trajectory picture with time *t* when *r* = 0.8, *K* = 10, *β*_1_ = 1, *a*_1_ = 1, *a*_2_ = 0.3, *d*_*h*_ = 0.3, *γ*_*h*_ = 0.06, *α*_*h*_ = 0.36, *p* = 0.007, *d*_*v*_ = 0.06, *β*_2_ = 0.26, and *N*_*v*_ = 2.

**Figure 7:**
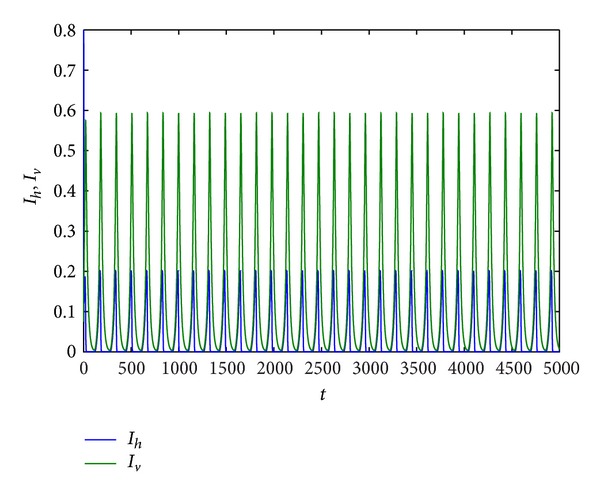
Trajectory picture with time *t* when *r* = 0.8, *K* = 10, *β*_1_ = 1, *a*_1_ = 1, *a*_2_ = 0.3, *d*_*h*_ = 0.6, *γ*_*h*_ = 0.06, *α*_*h*_ = 0.36, *p* = 0.007, *d*_*v*_ = 0.06, *β*_2_ = 0.26, and *N*_*v*_ = 2.

**Figure 8:**
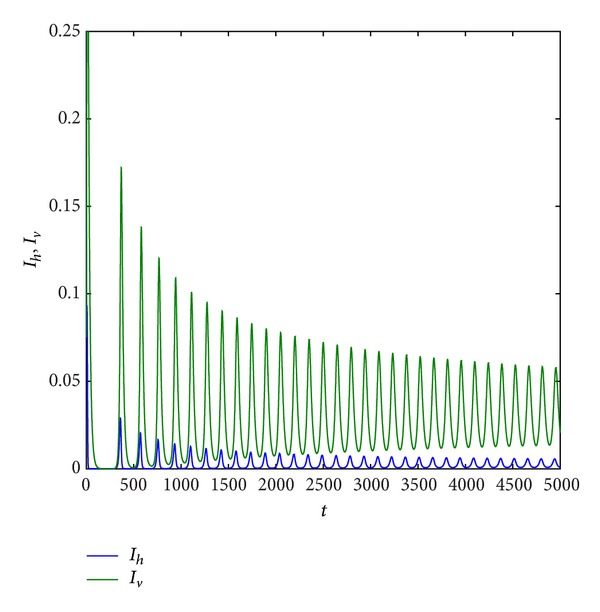
Trajectory picture with time *t* when *r* = 0.8, *K* = 10, *β*_1_ = 1, *a*_1_ = 1, *a*_2_ = 0.3, *d*_*h*_ = 0.7486, *γ*_*h*_ = 0.06, *α*_*h*_ = 0.36, *p* = 0.007, *d*_*v*_ = 0.06, *β*_2_ = 0.26, and *N*_*v*_ = 2.

**Figure 9:**
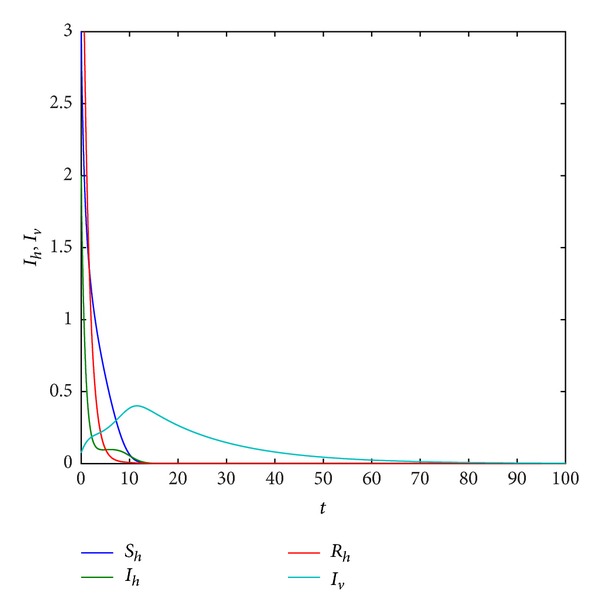
Trajectory picture with time *t* when *r* = 0.8, *K* = 10, *β*_1_ = 1, *a*_1_ = 1, *a*_2_ = 0.3, *d*_*h*_ = 0.8, *γ*_*h*_ = 0.06, *α*_*h*_ = 0.36, *p* = 0.007, *d*_*v*_ = 0.06, *β*_2_ = 0.26, and *N*_*v*_ = 2.

Take *r*, *K*, *β*_1_, *a*_1_ *γ*_*h*_,*α*_*h*_, *p*,*d*_*v*_,*β*_2_, and *N*_*v*0_ as the same with the above example and *a*_2_ = 0.3. We see how to change for trajectories *I*_*h*_ and *I*_*v*_ with time *t* when *d*_*h*_ = 0.2,0.3, 0.6,0.7486, and 0.8, respectively. From Figures 5 and 9, we can find that all of trajectories tend to an endemic equilibrium when *d*_*h*_ is smaller, and all of trajectories tend to an equilibrium (*S*_*h*_, *I*_*h*_, *R*_*h*_, *S*_*v*_, *I*_*v*_) = (0,0, 0, *N*_*v*0_, 0) when *d*_*h*_ is larger, that is, the host population will be distinct, and the vector population will be keep. If *d*_*h*_ = 0.3,0.6,0.7486, respectively, from Figures 6, 7, and 8, we find that system (3) has period solution. Furthermore, from these figures, we can see that the numbers of the infective host population and the infective vector population are on the decline with the natural death rate increase, but the rate of the decline for host population is much faster than that for vector population. Finally, the host population decays to zero.

## 4. Discussion

In previous literatures, the dynamic behaviors of models on vector-borne disease may have backward bifurcation and are not complex. In this paper, we consider the behaviors of a model with standard incidence rate and the logistic birth function for host population and the exponential birth rate for vector population. The rich dynamic behaviors are found. We find that the complex behaviors will not occur if the birth function is not logistic. Taking the natural death rate of host population as a bifurcation parameter, we find that the system may undergo a backward bifurcation, saddle-node bifurcation, Hopf bifurcation, Bogdanov-Takens bifurcation, and cusp bifurcation with the saturation parameter varying. We find that the natural death rate still is a key factor. If the natural death rate is higher, then the host population and the disease will die out; if it is smaller, then the host and vector population will coexist; that is, the disease will spread. If it is middle, the period solution will occur. In addition, the existence of equilibria of our model is different from previous model. If the incidence rate function is not affected by the number of vector populations, we find that there is always a unique positive equilibrium when the basic reproduction number *R*_0_ is less than one, and there are two endemic equilibria or no positive equilibrium when *R*_0_ is more than one. If the incidence rate function depends on the number of vector populations, we obtained that there may be two positive equilibria or no positive equilibrium when *R*_0_ < 1 and that there exist one or three endemic equilibria when *R*_0_ > 1 where the unique endemic one is always unstable.

## Figures and Tables

**Table 1:** The birth and incidence rates for different vector-borne disease models.

Disease	Literature	Birth		Incidence rate	
		*S*_*h*_	*S*_*v*_	Host	Vector
	[3]	λ_*h*_ *N*_*h*_	λ_*v*_ *N*_*v*_	$\frac{\beta_{h}I_{v}S_{h}}{N_{h}}$	$\frac{\beta_{v}I_{h}S_{v}}{N_{h}}$
Malaria	[5, 6]	Λ_*h*_ + λ_*h*_ *N*_*h*_	λ_*v*_ *N*_*v*_	$\frac{\beta_{h}I_{v}S_{h}}{\sigma_{v}N_{v}+\sigma_{h}N_{h}}$	$\frac{\beta_{v}I_{h}S_{v}}{\sigma_{v}N_{v}+\sigma_{h}N_{h}}$
	[7]	(1 − ϕ)Λ_*h*_	λ_*v*_ *N*_*v*_	$\frac{\beta_{h}I_{v}S_{h}}{N_{h}}$	$\frac{\beta_{v}I_{h}S_{v}}{N_{h}}$

	[8]	λ_*h*_ *N*_*h*_	Λ_*h*_	$\frac{\beta_{h}I_{v}S_{h}}{N_{h}+m}$	$\frac{\beta_{v}I_{h}S_{v}}{N_{h}+m}$
Dengue fever	[9]	λ_*h*_ *N*_*h*_	λ_*v*_ *N*_*v*_	$\frac{\beta_{h}I_{v}S_{h}}{N_{v}}$	$\frac{\beta_{v}I_{h}S_{v}}{N_{h}}$
	[10]	λ_*h*_ *N*_*h*_	λ_*v*_ *N*_*v*_	$\frac{\beta_{h}I_{v}S_{h}}{N_{h}+m}$	$\frac{\beta_{v}I_{h}S_{v}}{N_{h}+m}$

	[13]			$\frac{\beta_{1}I_{v}S_{h}}{N_{h}}$	$\frac{\beta_{v}I_{h}S_{v}}{N_{h}}$
WNV	[14]	Λ_*h*_	Λ_*v*_	$\frac{\beta_{h}I_{v}S_{h}}{N_{h}}$	$\frac{\beta_{v}I_{h}S_{v}}{N_{h}}$
	[15, 18]	Λ_*h*_	λ_*v*_ *N*_*v*_	$\frac{\beta_{h}I_{v}S_{h}}{N_{h}}$	$\frac{\beta_{v}I_{h}S_{v}}{N_{h}}$

**Table 2:** Parameters involved in the models for the dynamics of vector-borne diseases.

Parameters in the models	Vector	Host
Per capita birth rate	λ_*v*_	λ_*h*_
The fraction of the infective immigrants		ϕ
Number of times one mosquito would want to bite humans per unit time	σ_*h*_	
The maximum number of mosquito bites a human can have per unit time		σ_*v*_
Recruitment rate per unit of time	Λ_*v*_	Λ_*h*_
Per capita birth rate	ψ_*v*_	ψ_*h*_
Vertical transmission rate in new birth of vectors	*p*	
Natural death rate	*d*_*v*_	*d*_*h*_
Disease-induced death rate		α_*h*_
Recovery rate		γ_*h*_
Biting rate (the average number of bites per mosquito per day)	*b*	
Transmission probability (from infected vectors to host)		β_*h*_
Transmission probability (from infected host to vectors)	β_*v*_	
